# 2-Eth­oxy-6-{(*E*)-[(4-methyl­phen­yl)imino]­meth­yl}phenol

**DOI:** 10.1107/S1600536812024798

**Published:** 2012-06-13

**Authors:** Muhammad Ashraf Shaheen, M. Nawaz Tahir, Rana Muhammad Irfan, Shahid Iqbal, Saeed Ahmad

**Affiliations:** a Department of Chemistry, University of Sargodha, Sargodha, Pakistan; b Department of Physics, University of Sargodha, Sargodha, Pakistan; c Department of Chemistry, University of Engineering and Technology, Lahore 54890, Pakistan

## Abstract

The asymmetric unit of the title compound, C_16_H_17_NO_2_, contains two mol­ecules in which the dihedral angles between the 3-eth­oxy-2-hy­droxy­benzaldehyde and toluidine moieties are 16.87 (8) and 19.93 (6)°. *S*(6) rings are present in both mol­ecules due to intra­molecular O—H⋯N hydrogen bonds. In the crystal, one of the mol­ecules is dimerized with an inversion-generated partner, due to two C—H⋯O inter­actions. This generates an *R*_2_^2^(8) loop.

## Related literature

For related crystal structures, see: Albayrak *et al.* (2010 ▶); Özek *et al.* (2010 ▶).
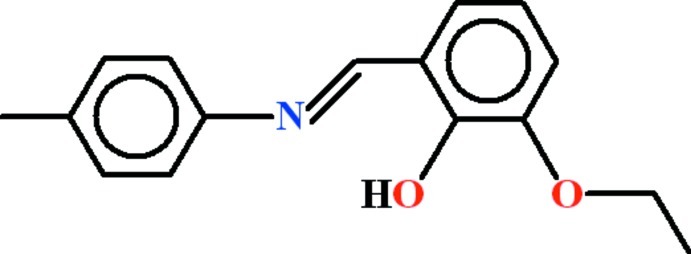


## Experimental

### 

#### Crystal data

C_16_H_17_NO_2_
*M* *_r_* = 255.31
Monoclinic, 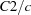

*a* = 29.5126 (11) Å
*b* = 6.8703 (3) Å
*c* = 28.2167 (13) Å
β = 102.986 (3)°
*V* = 5574.9 (4) Å^3^
*Z* = 16
Mo *K*α radiation
μ = 0.08 mm^−1^
*T* = 296 K
0.30 × 0.25 × 0.22 mm

#### Data collection

Bruker Kappa APEXII CCD diffractometer
Absorption correction: multi-scan (*SADABS*; Bruker, 2005 ▶) *T*_min_ = 0.975, *T*_max_ = 0.985
20559 measured reflections
5040 independent reflections
2075 reflections with *I* > 2σ(*I*)
*R*_int_ = 0.083

#### Refinement

*R*[*F* ^2^ > 2σ(*F* ^2^)] = 0.064
*wR*(*F* ^2^) = 0.181
*S* = 0.98
5040 reflections
346 parameters
H-atom parameters constrained
Δρ_max_ = 0.25 e Å^−3^
Δρ_min_ = −0.18 e Å^−3^

### 

Data collection: *APEX2* (Bruker, 2009 ▶); cell refinement: *SAINT* (Bruker, 2009 ▶); data reduction: *SAINT*; program(s) used to solve structure: *SHELXS97* (Sheldrick, 2008 ▶); program(s) used to refine structure: *SHELXL97* (Sheldrick, 2008 ▶); molecular graphics: *ORTEP-3 for Windows* (Farrugia, 1997 ▶) and *PLATON* (Spek, 2009 ▶); software used to prepare material for publication: *WinGX* (Farrugia, 1999 ▶) and *PLATON*.

## Supplementary Material

Crystal structure: contains datablock(s) global, I. DOI: 10.1107/S1600536812024798/hb6825sup1.cif

Structure factors: contains datablock(s) I. DOI: 10.1107/S1600536812024798/hb6825Isup2.hkl

Supplementary material file. DOI: 10.1107/S1600536812024798/hb6825Isup3.cml

Additional supplementary materials:  crystallographic information; 3D view; checkCIF report

## Figures and Tables

**Table 1:** Hydrogen-bond geometry (Å, °)

*D*—H⋯*A*	*D*—H	H⋯*A*	*D*⋯*A*	*D*—H⋯*A*
O1—H1⋯N1	0.82	1.86	2.584 (4)	147
O3—H3⋯N2	0.82	1.86	2.585 (3)	147
C24—H24*A*⋯O4^i^	0.96	2.59	3.470 (4)	153

Symmetry code: (i) 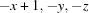.

**Fig. 1.**
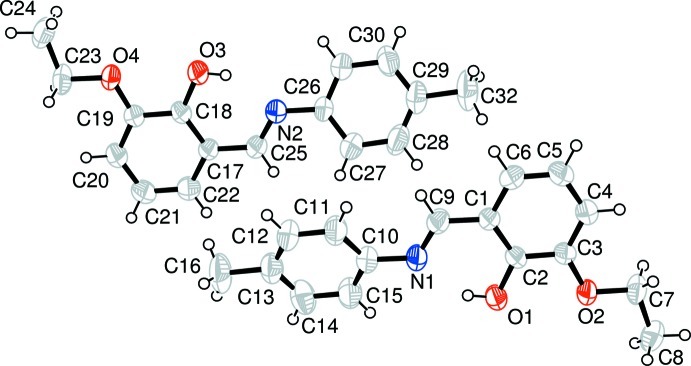
View of the title compound with displacement ellipsoids drawn at the 50% probability level.

**Fig. 2.**
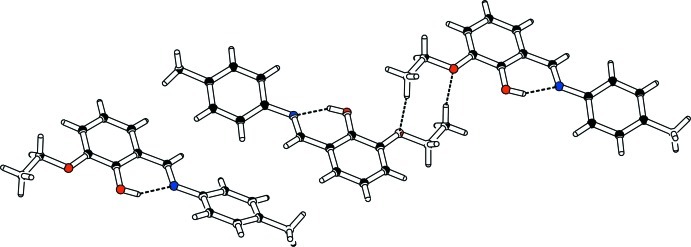
The partial packing, which shows that molecules form S(6) ring motif and one molecule is also dimerized with itself.

## supplementary crystallographic information

### Comment

The title compound (I), (Fig. 1) has been synthesized as a derivative for the complexation and other studies.

The crystal structures of 2-ethoxy-6-((phenylimino)methyl)phenol (Albayrak *et al.*, 2010) and (*E*)-2-ethoxy-6-[(4-ethoxyphenyl)iminomethyl]phenol (Özek *et al.*, 2010) have been published which are related to the title compound (I).

In (I), two molecules in the asymmetric unit are present, which differ slightly from each other geometrically. In one molecule, the group A (C1—C9/O1/O2) of 3-ethoxy-2-hydroxybenzaldehyde and group B (N1/C10—C16) of toluidine moieties are planar with r. m. s. deviation of 0.0270 Å and 0.0105 Å, respectively. The dihedral angle between A/B is 19.93 (6)°. In second molecule, the similar groups C (C18—C25/O3/O4) and D (N2/C26—C32) are also planar with r. m. s. deviation of 0.0184 Å and 0.0193 Å, respectively and the dihedral angle between C/D is 16.87 (8)°. In both molecules S(6) ring motif is present due to classical H–bonding of O—H···N type (Table 1, Fig. 2). The second molecule which is more planar is dimerized with itself from ethoxy groups with *R*_2_^2^(8) ring motif due to C—H···O type of H–bonding (Table 1, Fig. 2).

### Experimental

Equal molar ratio of 4-toluidine and 3-ethoxy-2-hydroxybenzaldehyde was refluxed in methanol for 2 h and orange prisms of (I) were obtained after 72 h by the slow evaporation at room temperature.

### Refinement

The H-atoms were positioned geometrically at C—H = 0.93—0.97 and O—H = 0.82 Å, respectively and included in the refinement as riding with *U*_iso_(H) = *xU*_eq_(C, O), where *x* = 1.5 for metyl H-atoms and *x* = 1.2 for all other H-atoms.

### Crystal data

**Table:**

C_16_H_17_NO_2_	*F*(000) = 2176
*M**_r_* = 255.31	*D*_x_ = 1.217 Mg m^−^^3^
Monoclinic, *C*2/*c*	Mo *K*α radiation, λ = 0.71073 Å
Hall symbol: -C 2yc	Cell parameters from 2075 reflections
*a* = 29.5126 (11) Å	θ = 1.8–25.3°
*b* = 6.8703 (3) Å	µ = 0.08 mm^−^^1^
*c* = 28.2167 (13) Å	*T* = 296 K
β = 102.986 (3)°	Prism, orange
*V* = 5574.9 (4) Å^3^	0.30 × 0.25 × 0.22 mm
*Z* = 16	

### Data collection

**Table:**

Bruker Kappa APEXII CCD diffractometer	5040 independent reflections
Radiation source: fine-focus sealed tube	2075 reflections with *I* > 2σ(*I*)
Graphite monochromator	*R*_int_ = 0.083
Detector resolution: 8.10 pixels mm^-1^	θ_max_ = 25.3°, θ_min_ = 1.8°
ω scans	*h* = −35→35
Absorption correction: multi-scan (*SADABS*; Bruker, 2005)	*k* = −8→8
*T*_min_ = 0.975, *T*_max_ = 0.985	*l* = −33→33
20559 measured reflections	

### Refinement

**Table:**

Refinement on *F*^2^	Secondary atom site location: difference Fourier map
Least-squares matrix: full	Hydrogen site location: inferred from neighbouring sites
*R*[*F*^2^ > 2σ(*F*^2^)] = 0.064	H-atom parameters constrained
*wR*(*F*^2^) = 0.181	*w* = 1/[σ^2^(*F*_o_^2^) + (0.071*P*)^2^] where *P* = (*F*_o_^2^ + 2*F*_c_^2^)/3
*S* = 0.98	(Δ/σ)_max_ = 0.001
5040 reflections	Δρ_max_ = 0.25 e Å^−^^3^
346 parameters	Δρ_min_ = −0.18 e Å^−^^3^
0 restraints	Extinction correction: *SHELXL97* (Sheldrick, 2008), Fc^*^=kFc[1+0.001xFc^2^λ^3^/sin(2θ)]^-1/4^
Primary atom site location: structure-invariant direct methods	Extinction coefficient: 0.0022 (8)

### Special details

**Table:**

Geometry. Bond distances, angles *etc*. have been calculated using the rounded fractional coordinates. All su's are estimated from the variances of the (full) variance-covariance matrix. The cell e.s.d.'s are taken into account in the estimation of distances, angles and torsion angles
Refinement. Refinement of *F*^2^ against ALL reflections. The weighted *R*-factor *wR* and goodness of fit *S* are based on *F*^2^, conventional *R*-factors *R* are based on *F*, with *F* set to zero for negative *F*^2^. The threshold expression of *F*^2^ > σ(*F*^2^) is used only for calculating *R*-factors(gt) *etc*. and is not relevant to the choice of reflections for refinement. *R*-factors based on *F*^2^ are statistically about twice as large as those based on *F*, and *R*- factors based on ALL data will be even larger.

### Fractional atomic coordinates and isotropic or equivalent isotropic displacement parameters (Å^2^)

**Table:**

	*x*	*y*	*z*	*U*_iso_*/*U*_eq_	
O1	0.10580 (7)	0.6814 (3)	0.19524 (10)	0.0684 (10)	
O2	0.01761 (7)	0.6161 (3)	0.18973 (9)	0.0585 (10)	
N1	0.18746 (9)	0.5514 (5)	0.19299 (10)	0.0550 (11)	
C1	0.12297 (10)	0.3411 (5)	0.19135 (12)	0.0461 (12)	
C2	0.09199 (11)	0.4939 (5)	0.19246 (12)	0.0473 (14)	
C3	0.04488 (11)	0.4545 (5)	0.18944 (12)	0.0468 (12)	
C4	0.02948 (11)	0.2647 (5)	0.18598 (12)	0.0574 (16)	
C5	0.06006 (12)	0.1118 (5)	0.18585 (13)	0.0629 (17)	
C6	0.10630 (11)	0.1513 (5)	0.18828 (12)	0.0580 (16)	
C7	−0.03139 (10)	0.5835 (6)	0.18454 (13)	0.0620 (16)	
C8	−0.05326 (11)	0.7791 (6)	0.18882 (13)	0.0721 (16)	
C9	0.17137 (11)	0.3781 (5)	0.19224 (12)	0.0527 (16)	
C10	0.23446 (11)	0.5901 (6)	0.19178 (12)	0.0533 (14)	
C11	0.27089 (11)	0.4584 (6)	0.20317 (13)	0.0670 (16)	
C12	0.31500 (12)	0.5126 (7)	0.19954 (15)	0.0753 (18)	
C13	0.32467 (12)	0.6948 (7)	0.18545 (13)	0.0665 (19)	
C14	0.28869 (14)	0.8279 (7)	0.17494 (14)	0.0794 (17)	
C15	0.24410 (12)	0.7749 (6)	0.17846 (14)	0.0710 (16)	
C16	0.37347 (8)	0.7535 (4)	0.18219 (13)	0.097 (2)	
O3	0.39693 (6)	0.0489 (3)	0.05775 (9)	0.0672 (10)	
O4	0.48472 (7)	0.1144 (3)	0.06181 (8)	0.0594 (10)	
N2	0.31507 (8)	0.1776 (4)	0.05970 (10)	0.0540 (11)	
C17	0.37945 (10)	0.3890 (5)	0.06123 (12)	0.0470 (12)	
C18	0.41055 (10)	0.2363 (5)	0.05998 (12)	0.0479 (12)	
C19	0.45777 (11)	0.2768 (5)	0.06204 (12)	0.0493 (14)	
C20	0.47255 (11)	0.4658 (6)	0.06428 (13)	0.0578 (16)	
C21	0.44186 (12)	0.6194 (5)	0.06494 (13)	0.0636 (17)	
C22	0.39611 (11)	0.5794 (5)	0.06345 (12)	0.0598 (16)	
C23	0.53338 (10)	0.1477 (6)	0.06427 (14)	0.0652 (16)	
C24	0.55535 (11)	−0.0499 (6)	0.06255 (14)	0.0765 (16)	
C25	0.33127 (10)	0.3496 (5)	0.06107 (12)	0.0519 (16)	
C26	0.26797 (10)	0.1379 (6)	0.06073 (12)	0.0520 (16)	
C27	0.23819 (12)	0.2710 (6)	0.07524 (13)	0.0686 (16)	
C28	0.19325 (13)	0.2127 (7)	0.07633 (14)	0.0766 (19)	
C29	0.17740 (12)	0.0278 (7)	0.06428 (14)	0.0694 (18)	
C30	0.20727 (12)	−0.1006 (6)	0.05007 (14)	0.0743 (17)	
C31	0.25229 (11)	−0.0466 (6)	0.04814 (14)	0.0666 (16)	
C32	0.12886 (12)	−0.0360 (7)	0.06682 (16)	0.101 (2)	
H1	0.13371	0.68676	0.19605	0.1027*	
H4	−0.00187	0.23879	0.18369	0.0689*	
H5	0.04949	−0.01611	0.18414	0.0755*	
H6	0.12678	0.04878	0.18786	0.0694*	
H7A	−0.03675	0.49650	0.20979	0.0740*	
H7B	−0.04466	0.52550	0.15315	0.0740*	
H8A	−0.04101	0.83189	0.22062	0.1082*	
H8B	−0.08634	0.76427	0.18387	0.1082*	
H8C	−0.04637	0.86579	0.16469	0.1082*	
H9	0.19133	0.27337	0.19226	0.0633*	
H11	0.26559	0.33328	0.21328	0.0803*	
H12	0.33892	0.42183	0.20695	0.0901*	
H14	0.29434	0.95355	0.16546	0.0954*	
H15	0.22034	0.86659	0.17166	0.0848*	
H16A	0.39353	0.75171	0.21409	0.1453*	
H16B	0.37283	0.88227	0.16884	0.1453*	
H16C	0.38490	0.66367	0.16157	0.1453*	
H3	0.36940	0.04294	0.05871	0.1006*	
H20	0.50366	0.49238	0.06538	0.0696*	
H21	0.45232	0.74747	0.06637	0.0763*	
H22	0.37562	0.68183	0.06394	0.0715*	
H23A	0.54759	0.21451	0.09421	0.0781*	
H23B	0.53738	0.22683	0.03703	0.0781*	
H24A	0.54192	−0.11186	0.03213	0.1145*	
H24B	0.54986	−0.12861	0.08882	0.1145*	
H24C	0.58822	−0.03496	0.06560	0.1145*	
H25	0.31139	0.45384	0.06200	0.0619*	
H27	0.24816	0.39701	0.08407	0.0823*	
H28	0.17328	0.30243	0.08558	0.0922*	
H30	0.19725	−0.22678	0.04151	0.0891*	
H31	0.27184	−0.13652	0.03824	0.0798*	
H32A	0.11408	0.06568	0.08117	0.1515*	
H32B	0.13071	−0.15157	0.08630	0.1515*	
H32C	0.11102	−0.06260	0.03461	0.1515*	

### Atomic displacement parameters (Å^2^)

**Table:**

	*U*^11^	*U*^22^	*U*^33^	*U*^12^	*U*^13^	*U*^23^
O1	0.0416 (14)	0.0433 (17)	0.122 (2)	−0.0037 (11)	0.0222 (14)	−0.0038 (15)
O2	0.0341 (14)	0.0511 (17)	0.0919 (19)	0.0002 (11)	0.0178 (11)	−0.0033 (14)
N1	0.0366 (17)	0.054 (2)	0.075 (2)	0.0002 (15)	0.0137 (13)	0.0008 (18)
C1	0.040 (2)	0.040 (2)	0.059 (2)	0.0006 (17)	0.0126 (15)	−0.0014 (18)
C2	0.041 (2)	0.038 (2)	0.063 (3)	−0.0057 (16)	0.0121 (15)	−0.0018 (18)
C3	0.043 (2)	0.038 (2)	0.060 (2)	−0.0002 (18)	0.0128 (15)	−0.0001 (19)
C4	0.044 (2)	0.052 (3)	0.077 (3)	−0.0044 (19)	0.0155 (17)	−0.004 (2)
C5	0.057 (3)	0.042 (3)	0.090 (3)	−0.0063 (19)	0.017 (2)	−0.001 (2)
C6	0.052 (2)	0.047 (3)	0.076 (3)	0.0087 (18)	0.0163 (17)	−0.001 (2)
C7	0.035 (2)	0.076 (3)	0.077 (3)	0.0011 (18)	0.0166 (17)	0.006 (2)
C8	0.045 (2)	0.085 (3)	0.088 (3)	0.016 (2)	0.0186 (18)	0.003 (2)
C9	0.043 (2)	0.050 (3)	0.065 (3)	0.0067 (18)	0.0122 (16)	0.004 (2)
C10	0.041 (2)	0.061 (3)	0.059 (2)	−0.0005 (19)	0.0138 (16)	0.001 (2)
C11	0.046 (2)	0.066 (3)	0.090 (3)	0.000 (2)	0.0172 (19)	0.007 (2)
C12	0.042 (2)	0.088 (4)	0.097 (3)	0.003 (2)	0.018 (2)	−0.007 (3)
C13	0.049 (3)	0.093 (4)	0.060 (3)	−0.010 (2)	0.0175 (19)	−0.008 (3)
C14	0.063 (3)	0.087 (3)	0.089 (3)	−0.017 (3)	0.019 (2)	0.019 (3)
C15	0.051 (2)	0.070 (3)	0.092 (3)	−0.001 (2)	0.0161 (19)	0.014 (3)
C16	0.052 (2)	0.150 (5)	0.095 (3)	−0.025 (3)	0.031 (2)	−0.005 (3)
O3	0.0427 (14)	0.0433 (17)	0.119 (2)	−0.0046 (12)	0.0256 (12)	0.0009 (15)
O4	0.0348 (14)	0.0553 (18)	0.0910 (19)	−0.0002 (11)	0.0202 (12)	−0.0012 (14)
N2	0.0412 (18)	0.048 (2)	0.073 (2)	−0.0004 (14)	0.0135 (13)	−0.0014 (17)
C17	0.038 (2)	0.039 (2)	0.064 (2)	0.0015 (16)	0.0112 (15)	0.0012 (19)
C18	0.042 (2)	0.042 (2)	0.061 (2)	−0.0069 (17)	0.0141 (16)	0.0009 (19)
C19	0.040 (2)	0.044 (3)	0.065 (2)	−0.0029 (18)	0.0142 (16)	−0.003 (2)
C20	0.045 (2)	0.051 (3)	0.077 (3)	−0.0080 (19)	0.0131 (17)	0.000 (2)
C21	0.060 (3)	0.044 (3)	0.087 (3)	−0.008 (2)	0.017 (2)	0.000 (2)
C22	0.053 (2)	0.047 (3)	0.079 (3)	0.0012 (19)	0.0142 (18)	0.003 (2)
C23	0.035 (2)	0.083 (3)	0.080 (3)	−0.0020 (19)	0.0182 (17)	0.007 (2)
C24	0.046 (2)	0.095 (3)	0.090 (3)	0.015 (2)	0.0184 (19)	−0.005 (3)
C25	0.041 (2)	0.047 (3)	0.067 (3)	0.0054 (17)	0.0106 (16)	0.001 (2)
C26	0.034 (2)	0.059 (3)	0.063 (3)	0.0003 (18)	0.0109 (16)	−0.004 (2)
C27	0.053 (2)	0.075 (3)	0.083 (3)	0.002 (2)	0.0261 (19)	−0.011 (2)
C28	0.048 (3)	0.101 (4)	0.087 (3)	0.006 (2)	0.028 (2)	−0.014 (3)
C29	0.043 (2)	0.103 (4)	0.065 (3)	−0.007 (2)	0.0181 (18)	0.000 (3)
C30	0.051 (3)	0.086 (3)	0.088 (3)	−0.017 (2)	0.020 (2)	−0.009 (3)
C31	0.049 (2)	0.067 (3)	0.088 (3)	−0.003 (2)	0.0244 (19)	−0.005 (2)
C32	0.052 (3)	0.158 (5)	0.101 (4)	−0.023 (3)	0.035 (2)	−0.003 (3)

### Geometric parameters (Å, º)

**Table:**

O1—C2	1.348 (4)	C12—H12	0.9300
O2—C3	1.372 (4)	C14—H14	0.9300
O2—C7	1.438 (4)	C15—H15	0.9300
O1—H1	0.8200	C16—H16C	0.9600
O3—C18	1.346 (4)	C16—H16A	0.9600
O4—C19	1.371 (4)	C16—H16B	0.9600
O4—C23	1.440 (4)	C17—C22	1.394 (5)
O3—H3	0.8200	C17—C25	1.447 (4)
N1—C9	1.280 (5)	C17—C18	1.400 (5)
N1—C10	1.420 (4)	C18—C19	1.409 (5)
N2—C25	1.272 (4)	C19—C20	1.367 (5)
N2—C26	1.423 (4)	C20—C21	1.394 (5)
C1—C6	1.390 (5)	C21—C22	1.369 (5)
C1—C2	1.397 (5)	C23—C24	1.510 (6)
C1—C9	1.446 (5)	C26—C31	1.369 (6)
C2—C3	1.400 (5)	C26—C27	1.392 (5)
C3—C4	1.377 (5)	C27—C28	1.392 (5)
C4—C5	1.386 (5)	C28—C29	1.370 (7)
C5—C6	1.378 (5)	C29—C32	1.515 (5)
C7—C8	1.507 (6)	C29—C30	1.369 (6)
C10—C15	1.372 (6)	C30—C31	1.392 (5)
C10—C11	1.387 (5)	C20—H20	0.9300
C11—C12	1.380 (5)	C21—H21	0.9300
C12—C13	1.363 (7)	C22—H22	0.9300
C13—C14	1.382 (6)	C23—H23A	0.9700
C13—C16	1.518 (5)	C23—H23B	0.9700
C14—C15	1.390 (6)	C24—H24A	0.9600
C4—H4	0.9300	C24—H24B	0.9600
C5—H5	0.9300	C24—H24C	0.9600
C6—H6	0.9300	C25—H25	0.9300
C7—H7A	0.9700	C27—H27	0.9300
C7—H7B	0.9700	C28—H28	0.9300
C8—H8C	0.9600	C30—H30	0.9300
C8—H8A	0.9600	C31—H31	0.9300
C8—H8B	0.9600	C32—H32A	0.9600
C9—H9	0.9300	C32—H32B	0.9600
C11—H11	0.9300	C32—H32C	0.9600
			
C3—O2—C7	116.8 (3)	H16A—C16—H16B	109.00
C2—O1—H1	109.00	C13—C16—H16A	109.00
C19—O4—C23	116.3 (3)	C13—C16—H16B	109.00
C18—O3—H3	110.00	C18—C17—C22	118.6 (3)
C9—N1—C10	122.3 (3)	C18—C17—C25	120.6 (3)
C25—N2—C26	122.7 (3)	C22—C17—C25	120.8 (3)
C2—C1—C6	118.8 (3)	O3—C18—C19	118.2 (3)
C2—C1—C9	121.1 (3)	C17—C18—C19	119.9 (3)
C6—C1—C9	120.1 (3)	O3—C18—C17	121.8 (3)
O1—C2—C3	118.1 (3)	O4—C19—C20	126.4 (3)
O1—C2—C1	121.9 (3)	C18—C19—C20	119.5 (3)
C1—C2—C3	120.0 (3)	O4—C19—C18	114.1 (3)
C2—C3—C4	119.7 (3)	C19—C20—C21	121.2 (3)
O2—C3—C4	125.6 (3)	C20—C21—C22	119.1 (3)
O2—C3—C2	114.8 (3)	C17—C22—C21	121.7 (3)
C3—C4—C5	120.9 (3)	O4—C23—C24	106.6 (3)
C4—C5—C6	119.3 (3)	N2—C25—C17	122.4 (3)
C1—C6—C5	121.4 (3)	N2—C26—C31	116.6 (3)
O2—C7—C8	107.0 (3)	C27—C26—C31	119.1 (3)
N1—C9—C1	121.7 (3)	N2—C26—C27	124.3 (3)
C11—C10—C15	118.0 (3)	C26—C27—C28	119.0 (4)
N1—C10—C15	116.3 (3)	C27—C28—C29	122.4 (4)
N1—C10—C11	125.7 (4)	C28—C29—C30	117.6 (4)
C10—C11—C12	120.1 (4)	C28—C29—C32	122.0 (4)
C11—C12—C13	122.2 (4)	C30—C29—C32	120.4 (4)
C12—C13—C14	118.0 (4)	C29—C30—C31	121.5 (4)
C14—C13—C16	120.3 (4)	C26—C31—C30	120.4 (3)
C12—C13—C16	121.7 (3)	C19—C20—H20	119.00
C13—C14—C15	120.4 (4)	C21—C20—H20	119.00
C10—C15—C14	121.3 (4)	C20—C21—H21	120.00
C5—C4—H4	120.00	C22—C21—H21	120.00
C3—C4—H4	120.00	C17—C22—H22	119.00
C6—C5—H5	120.00	C21—C22—H22	119.00
C4—C5—H5	120.00	O4—C23—H23A	110.00
C5—C6—H6	119.00	O4—C23—H23B	110.00
C1—C6—H6	119.00	C24—C23—H23A	110.00
O2—C7—H7B	110.00	C24—C23—H23B	110.00
H7A—C7—H7B	109.00	H23A—C23—H23B	109.00
C8—C7—H7A	110.00	C23—C24—H24A	109.00
C8—C7—H7B	110.00	C23—C24—H24B	109.00
O2—C7—H7A	110.00	C23—C24—H24C	109.00
H8A—C8—H8B	109.00	H24A—C24—H24B	109.00
H8A—C8—H8C	109.00	H24A—C24—H24C	109.00
H8B—C8—H8C	109.00	H24B—C24—H24C	109.00
C7—C8—H8A	109.00	N2—C25—H25	119.00
C7—C8—H8B	109.00	C17—C25—H25	119.00
C7—C8—H8C	109.00	C26—C27—H27	120.00
N1—C9—H9	119.00	C28—C27—H27	120.00
C1—C9—H9	119.00	C27—C28—H28	119.00
C12—C11—H11	120.00	C29—C28—H28	119.00
C10—C11—H11	120.00	C29—C30—H30	119.00
C13—C12—H12	119.00	C31—C30—H30	119.00
C11—C12—H12	119.00	C26—C31—H31	120.00
C13—C14—H14	120.00	C30—C31—H31	120.00
C15—C14—H14	120.00	C29—C32—H32A	109.00
C10—C15—H15	119.00	C29—C32—H32B	109.00
C14—C15—H15	119.00	C29—C32—H32C	109.00
C13—C16—H16C	109.00	H32A—C32—H32B	109.00
H16A—C16—H16C	109.00	H32A—C32—H32C	109.00
H16B—C16—H16C	109.00	H32B—C32—H32C	109.00
			
C7—O2—C3—C2	177.4 (3)	C10—C11—C12—C13	0.8 (6)
C7—O2—C3—C4	−1.9 (5)	C11—C12—C13—C14	0.5 (6)
C3—O2—C7—C8	176.8 (3)	C11—C12—C13—C16	179.2 (4)
C19—O4—C23—C24	−179.0 (3)	C16—C13—C14—C15	−179.3 (3)
C23—O4—C19—C20	0.1 (5)	C12—C13—C14—C15	−0.5 (6)
C23—O4—C19—C18	−179.5 (3)	C13—C14—C15—C10	−0.8 (6)
C9—N1—C10—C15	161.4 (3)	C22—C17—C18—O3	−179.9 (3)
C10—N1—C9—C1	−177.3 (3)	C22—C17—C18—C19	1.4 (5)
C9—N1—C10—C11	−19.1 (5)	C25—C17—C18—O3	1.2 (5)
C25—N2—C26—C31	165.7 (3)	C25—C17—C18—C19	−177.6 (3)
C25—N2—C26—C27	−16.7 (5)	C18—C17—C22—C21	−0.7 (5)
C26—N2—C25—C17	178.4 (3)	C25—C17—C22—C21	178.2 (3)
C9—C1—C2—O1	−1.2 (5)	C18—C17—C25—N2	−0.2 (5)
C9—C1—C2—C3	177.2 (3)	C22—C17—C25—N2	−179.1 (3)
C6—C1—C2—C3	−1.4 (5)	O3—C18—C19—O4	−0.4 (4)
C6—C1—C2—O1	−179.8 (3)	O3—C18—C19—C20	179.9 (3)
C2—C1—C9—N1	−1.6 (5)	C17—C18—C19—O4	178.4 (3)
C2—C1—C6—C5	0.7 (5)	C17—C18—C19—C20	−1.3 (5)
C9—C1—C6—C5	−178.0 (3)	O4—C19—C20—C21	−179.1 (3)
C6—C1—C9—N1	177.0 (3)	C18—C19—C20—C21	0.5 (5)
C1—C2—C3—O2	−178.5 (3)	C19—C20—C21—C22	0.2 (5)
O1—C2—C3—O2	−0.1 (4)	C20—C21—C22—C17	−0.1 (5)
O1—C2—C3—C4	179.3 (3)	N2—C26—C27—C28	−177.7 (3)
C1—C2—C3—C4	0.8 (5)	C31—C26—C27—C28	−0.3 (5)
O2—C3—C4—C5	179.8 (3)	N2—C26—C31—C30	177.3 (3)
C2—C3—C4—C5	0.5 (5)	C27—C26—C31—C30	−0.3 (6)
C3—C4—C5—C6	−1.3 (5)	C26—C27—C28—C29	1.0 (6)
C4—C5—C6—C1	0.7 (5)	C27—C28—C29—C30	−1.0 (6)
N1—C10—C15—C14	−178.5 (3)	C27—C28—C29—C32	178.4 (4)
C15—C10—C11—C12	−2.0 (5)	C28—C29—C30—C31	0.4 (6)
N1—C10—C11—C12	178.6 (3)	C32—C29—C30—C31	−179.0 (4)
C11—C10—C15—C14	2.0 (6)	C29—C30—C31—C26	0.3 (6)

### Hydrogen-bond geometry (Å, º)

**Table:**

*D*—H···*A*	*D*—H	H···*A*	*D*···*A*	*D*—H···*A*
O1—H1···N1	0.82	1.86	2.584 (4)	147
O3—H3···N2	0.82	1.86	2.585 (3)	147
C24—H24*A*···O4^i^	0.96	2.59	3.470 (4)	153

Symmetry code: (i) −*x*+1, −*y*, −*z*.

## References

[bb1] Albayrak, Ç., Koşar, B., Demir, S., Odabaşoĝlu, M. & Büyükgüngör, O. (2010). *J. Mol. Struct.* **963**, 211–218.

[bb2] Bruker (2005). *SADABS* Bruker AXS Inc., Madison, Wisconsin, USA.

[bb3] Bruker (2009). *APEX2* and *SAINT* Bruker AXS Inc., Madison, Wisconsin, USA.

[bb4] Farrugia, L. J. (1997). *J. Appl. Cryst.* **30**, 565.

[bb5] Farrugia, L. J. (1999). *J. Appl. Cryst.* **32**, 837–838.

[bb6] Özek, A., Koşar, B., Albayrak, Ç. & Büyükgüngör, O. (2010). *Acta Cryst.* E**66**, o684. 10.1107/S1600536810006434 PMC2983578 21580427

[bb7] Sheldrick, G. M. (2008). *Acta Cryst.* A**64**, 112–122. 10.1107/S0108767307043930 18156677

[bb8] Spek, A. L. (2009). *Acta Cryst.* D**65**, 148–155. 10.1107/S090744490804362X PMC2631630 19171970

